# Use of Antimicrobial Irrigation and Incidence of Capsular Contracture in Breast Augmentation and Immediate Implant-Based Breast Reconstruction

**DOI:** 10.1007/s00266-023-03453-5

**Published:** 2023-07-06

**Authors:** Giuzio Federica, Fabrizio Tommaso, Catalano Alessia, Ceccarini Agostino, Bodog Florian, Giuliani Antonio, Massariello Domenico Nicola, Raweh Abdallah, Saturnino Carmela, Svolacchia Lorenzo, Brongo Sergio

**Affiliations:** 1 Department of Sciences, University of Basilicata, Potenza, Italy; 2 Spinoff TNcKILLERS s.r.l, Ateneo Lucano street 10, 85100 Potenza, Italy; 3 Department of Plastic Surgery, IRCCS, Referral Cancer Center of Basilicata, Rionero in Vulture, PZ Italy; 4 Department of Pharmacy‐Drug Sciences, University of Bari “Aldo Moro”, Bari, Italy; 5 U.O.C. Primary Care and Territorial Health, Social and Health Department, State Hospital, Falciano, Republic of San Marino; 6 Department of Surgery, Faculty of Medicine and Pharmacy, University of Oradea, Oradea, Romania; 7 U.O.C. of General and Emergency Surgery A.O.R. “San Carlo”, Potenza, Basilicata Italy; 8 U.O.S.D of Plastic Surgery A.O.R “San Carlo”, Potenza, Italy; 9 Cardiac Surgery Department, Yas Clinic, Abu Dhabi, United Arab Emirates; 10 Departments of Medical-Surgical Sciences and Biotechnologies, La Sapienza University, Rome, Italy; 11 Department of Plastic Surgery, University of Salerno, Campania, Italy

**Keywords:** Capsular contracture, Breast implant, Breast reconstruction, Topical antibiotics, Surgical site infections

## Abstract

Capsular contracture (CC) is one of the most common complications of implant-based breast reconstruction or augmentation surgery. Common risk factors of CC include biofilm, surgical site infections, history of prior CC or fibrosis, history of radiation therapy, and implant characteristics. Though bacterial contamination of breast protheses is associated with adverse sequelae, there are not universally accepted guidelines and limited best practice recommendations for antimicrobial breast pocket irrigation. Despite advanced molecular biology, the exact mechanism of this complication is not fully understood. Interventions that decrease the rate of CC include antibiotic prophylaxis or irrigation, acellular dermal matrix, leukotriene inhibitors, surgical techniques, and others. However, there is inconsistent evidence supporting these risk factors, and the current data was based on broad heterogeneous studies. The objective of this review was to provide a summary of the current data of contributing risk factors as well as preventative and treatment measures for CC.

*Level of Evidence III* This journal requires that authors assign a level of evidence to each article. For a full description of these evidence-based medicine ratings, please refer to the Table of Contents or the online Instructions to Authors http://www.springer.com/00266

## Introduction

Breast cancer is the most frequently diagnosed cancer in women around the world, and up to 41% of patients who undergo mastectomy receive breast reconstruction [1]. Breast implant has been present since the 1960s, and 65% of reconstruction surgery is implant-based in the United States [2]. The main goals of breast reconstruction are to reshape the breast due to tissue loss following breast cancer; to revise and fix the previous reconstruction surgery; and to augment the breast volume for cosmetic purposes. Along with its advantages for physical and psychological satisfaction given for the patients, complication rates are high following implant-based breast reconstruction especially for capsular contracture.

CC is a distressing complication of breast implant surgery and often requires revision operation. Up to half of the patients develop CC, and 30% of them suffer from CC with Baker rates III and IV following implant-based breast reconstruction [3, 4].

Risk factors found to be associated with CC include previous capsular fibrosis, radiation therapy, contamination with biofilm-producing bacteria, surgical site infections (SSI), and immune response to the foreign material [3, 5]. The expression of toll-like receptor 4 is also seen in peri-implant tissue fibrosis and may play a role in myofibroblast differentiation to induce CC development [6]. The exact mechanism of the pathophysiology of CC formation is still unknown. An infection has been linked with the formation of CC extensively. Generally, breast surgery is considered to be a clean surgery but the postoperative SSI rate rises by 2–2.9% in augmentation and is the most common cause of readmission [7, 8]. The common organisms identified are Staphylococcus epidermidis and S. aureus, Escherichia, Pseudomonas, Propionibacterium, and Corynebacterium [9, 10].

This study aims to review the risk factors associated with CC and to outline the available preventative and treatment measures to reduce the rate of CC.

## Methods

A comprehensive literature review was completed to create a list of pocket irrigation solutions currently described in the literature. We identified 413 publications total from PubMed search and excluded 397 publications due to not corresponding to our topic. Fifteen studies were ultimately selected and included in our review.

## Results

Preoperative skin antiseptic agents are known to reduce postoperative complications. CC rate is reduced with povidone-iodine and antimicrobial irrigations [11]; however, chlorhexidine gluconate was found to be more effective than povidone-iodine for reduction of biofilm-related CC [12].

In 1986, Burkhardt et al. described the relationship between bacterial contamination and implant-related comorbidity; they demonstrated that the use of local antimicrobial agents in and around retromammary implants improved surgical outcomes, with an incidence of capsular contracture 7 times less than the control group [13].

Pocket irrigation with Betadine (Purdue Frederick, Stamford, Conn.) became a standard in practice as the literature increasingly supported the role of microrganisms as the basis of capsular contracture. In 2000, the FDA deemed the use of Betadine for breast pocket irrigation contraindicated, citing that exposure may lead to early implant failure [14].

Following the 2000 FDA ban on Betadine, Adams et al. proposed a triple antibiotic solution (TAS) composed of 50,000 U Bacitracin, 1 g Ancef, and 80 mg Gentamicin and recommended a pocket contact time of 5 minutes. Subsequent studies demonstrated that TAS is associated with a rate of capsular contracture 4 to 5 times lower in breast augmentation patients [15].

The FDA subsequently removed the warning on the use of Betadine with breast implants in 2017 [16]. Consequently, in early 2018, Jewell and Adams [17] updated a 14-point plan originally published in 2013 designed to decrease bacterial bioburden in breast implant surgery, including pocket irrigation with TAS, TAS + Betadine, or ≥50% Betadine [18]. Adams suggests the need for consensus among plastic surgeons regarding pocket irrigation and further recommends that surgeons “should simply utilize the proven ingredients and ratios as recommended [19]”. Table 1 summarizes the mechanisms of action of individual antimicrobial agents according to the main guidelines of the American Society of Plastic Surgery [20].

**Table 1.** Antimicrobial agents preferred among american society of plastic surgery (ASPS) survey respondents

Antimicrobial agents	Mechanism of action	Spectrum
Benzalkonium chloride	Degrades cell wall causing leakage of cellular contents; surfactant properties; solution uses mechanical debridement with a pulse lavage device	More effective against Gram positive than Gram negative
0.1%/0.1% Polyhexanide/Betaine soap		
0.05% aqueous chlorhexidine Gluconate	Biguanide that disrupts cell walls and precipitates cellular proteins; binds to cell walls and alters osmotic equilibrium	Gram-negative and Gram-positive
0.05% chlorhexidine gluconate soap	At physiological pH, chlorhexidine slats dissociate and release positively charged chlorhexidine cation which binds to negatively charged bacterial cell walls	Broad antimicrobial coverage
Hydrogen peroxide	Oxidant; causes tissue toxicity via corrosive damage, oxygen gas formation, and lipid peroxidation	Broad coverage against viruses, bacteria, yeasts, and bacterial spores
Iodine-containing salts 10% povidone-iodine: I-PVP	Causes protein denaturation and precipitation of bacteria; toxic toward human fibroblasts	Viruses, bacteria, spores, fungi, and protozoa
Ammonium chlorides (bleaches) 0.25% sodium hypochlorite	Increases pH and interferes with cytoplasmic membrane integrity; interferes with cellular metabolism and phospholipid degradation	Broad coverage; dose-dependent toxicity against macrophages
0.4% sodium oxychlorosene	Oxidation and hypochlorination, and thereby destruction, of protoplasmic contents	Broad coverage
0.025% hypochlorous acid	Replicates oxidative burst that occurs in white blood cells with the release of hypochlorous acid	Broad coverage against Gram-positive and Gram-negative bacteria and fungi
Cefazolin Gentamicin (Anacef)	Inhibits cell wall synthesis Binds the 30S subunit of bactericidal ribosome, interrupting protein synthesis	Broad coverage against Gram-negative and Gram-positive organisms; concentration dependent
Bacitracin	Disrupts bacterial cell wall synthesis and inhibits cell enzymes	Most Gram-positive organisms
Polymyxin B	Binds to cell membrane and alters structure, making it permeable	Resistant Gram-negative microbes except Proteus and Neisseria genera
Vancomycin	Inhibits cell wall synthesis	Gram-positive bacteria

Staphylococci species are the most common axillary flora, and antibiotics targeted at these species do not show a significant impact on SSI [21]. Preoperative prophylaxis has not significantly reduced SSI in breast cancer surgery [22], and prolonged postoperative antibiotic prophylaxis also has not shown to decrease implant loss or highly virulent infections [23]. Patient compliance plays an important role in preventing SSI, and medication noncompliance doubles the risk of infection in breast surgery [24]. In primary breast augmentation, most organisms in acute infections are Gram-positives and are adequately covered by a single dose of IV cephalosporin; clindamycin or vancomycin is recommended in individuals with β-lactam allergies. The antibiotic is broadened with fluoroquinolones or vancomycin in late infections or secondary surgeries due to mixed organisms with both Gram-positives and Gram-negatives [8].

Post-mastectomy radiation therapy leads to higher rates of CC [25]. Several studies report that patients who had radiation therapy are more likely to experience reconstruction failure due to complications. The expression of Thy1 (CD90), which has an important role in scar tissue formation, is shown to be increased by radiation; thus, targeting the Thy1 receptor may decrease the rate of radiation-induced fibroproliferation of capsular tissue [26]. Muscle fibrosis is another possible contributor to CC in irradiated patients with subpectoral implant placement vs. prepectoral implant placement [27].

Breast implant characteristics especially implant surface seem to play a role in CC. Studies have analyzed that smooth implants, compared to textured implants, are significantly associated with CC, and the choice of the textured implant may reduce the risk of CC [28]. Moreover, microtextured implants may have lower rates of CC compared to macrotextured implants. However, macrotextured implants have been associated with increased risk of anaplastic large-cell lymphoma (ALCL) significantly compared to smooth or microtextured implants [29, 30]. Breast implant-associated ALCL is a rare complication and may have an infectious cause as seen by the bacterial biofilm on the implant [31].

LTE (Leukotriene) antagonists have been known to prevent and treat CC. Multiple studies have found that the patients who used LTE antagonists, either montelukast or zafirlukast, have significantly decreased rate of CC compared to the control group [32, 33]. Although there is a short-term benefit in CC reduction rates with the use of LTE antagonists, its long-term side effects such as liver damage are not known in depth [34].

## Discussion

The purpose of this review was to analyze the risk factors, etiology, and preventions for CC and to provide recommendations according to the current literature. The routine use of antimicrobial pocket irrigation and implant soaking agents, inframammary fold incision technique, and submuscular implant placement have led to decreased rates of capsular contracture [28]. The most preferred incision location among all survey respondents was within the inframammary fold. This finding is supported by the literature which demonstrates that the inframammary approach has been associated with a statistically significant reduction in capsular contracture [35]. Also in accordance with the literature, the most favored implant placement was in a submuscular pocket. This is likely due to its association with lower rates of infection and capsular contracture as predicted by Burkhardt et al. in 1986 [13]. Despite the strong association between bacteria and surgical complications, there appear to be no universally accepted, evidence-based best practice guidelines regarding antimicrobial breast pocket irrigation practices and only a grade D (level V evidence) guidelines for perioperative antibiotic practices. The current literature regarding pocket irrigation presents a confusing and conflicting picture regarding recommended solutions [36]. Much of the recent literature on pocket irrigation and implant soaking practices supports the use of TAS. However, studies have identified superior efficacy of Betadine-containing irrigations. Additionally, 1 study found non-Betadine containing TAS and 0.05% chlorhexidine to be most effective [37, 38]. Despite support in the literature for the use of TAS and TAS + Betadine, only 63% of respondents utilize TAS, TAS + Betadine (“Betadine Quadruple”), or TAS + Betadine without Bacitracin (“Betadine Triple”) as a pocket irrigant in their cosmetic cases. In all, over 35 distinct pocket irrigation solutions were identified among ASPS members during augmentation mammaplasty [39, 40]. Fisher reintroduced the concept of time-dependent efficacy of irrigation solutions in breast aug-mentation [39]. Pharmacologically, the efficacy of some antibiotics, such as Ancef, is dependent on time rather than concentration, such as with Gentamycin [41–44]. In contrast to reconstructive and implant-salvage procedures where evacuative drain placement is routine, pocket irrigation dwell time may be less significant in augmentation mammaplasty when the solution is left in the pocket, as suggested by Adams, thereby achieving prolonged exposure times [45, 46].

## Conclusions

CC is most likely to be multifactorial, and the exact mechanism of pathogenesis of CC formation is unknown. The available evidence on risk factors associated with CC is weak and inconclusive. Our review suggests that infectious cause may be the strongest risk factor of CC etiology, and further studies on this aspect are required. The current literature data on prevention and treatment of CC is heterogeneous, and results are controversial. Greater efforts in developing modern imaging and technologies will continue to provide advanced tools to understand the pathophysiology of CC in depth and further develop preventative and treatment interventions. According to the current literature, the incidence of SSI and CC has decreased with antibiotic prophylaxis, textured implant, ADM, leukotriene antagonists, and an open capsulotomy; however, these interventions have not been proved. The important question to be addressed should be more focused on the pathogenesis of CC, which has been debatable. It is important to carefully evaluate the pathophysiological mechanism underlying capsular contracture. Etoricoxib (cox- 2 inhibitor) and zafirlukast (leukotriene inhibitor) have proven effective overall in prevent and reduce periprosthetic capsular contracture. The activity of cox-2 inhibitors was greater than leukotriene inhibitors. However, the potential cardiotoxicity of the former, as opposed to the very low side effects of the latter make zafirlukast preferred as the drug of first choice in post oncological breast reconstruction with prosthetic materials. All the patients treated with Accoleit® -zafirlukast showed no intolerance or reaction no adverse drug, this in support of the relative safety of one of its off label use [43]. It is currently necessary to investigate how to prevent capsular contracture or to minimize the risk of onset. At present, further studies and investigations of a pharmacological, surgical and pathological nature need to be carried out.
